# Older Drivers: Attention, Habits and Memory

**DOI:** 10.1093/geroni/igaf122.3986

**Published:** 2025-12-31

**Authors:** Laura Hemmy, Katelyn Schwieters, Marshall Mabry, William Kessler, Brian Davis, Nichole Morris

**Affiliations:** University of Minnesota, Minneapolis, Minnesota, United States; University of Minnesota, Minneapolis, Minnesota, United States; University of Minnesota, Minneapolis, Minnesota, United States; University of Minnesota, Minneapolis, Minnesota, United States; University of Minnesota, Minneapolis, Minnesota, United States; University of Minnesota, Minneapolis, Minnesota, United States

## Abstract

Older drivers are at increased risk for negative outcomes and generating knowledge to promote safe driving and when to consider retirement from driving are of the utmost importance. This study evaluated how demographics and cognitive state are related to driver habits and attention. A sample of community dwelling older adults (n = 111) without global cognitive impairment on the Minnesota Cognitive Acuity Screen completed baseline assessments before the initiation of a driver safety intervention. Participants reported as 61% female, mean age 69.1 (SD 6.2), 15.9 mean years education (SD 2.4), and 66% retired. 19% reported living in an urban setting, 47% suburban, and 34% rural. Self-reported measures included the Driving and Riding Avoidance Scale (DRAS), Cognitive Failures Questionnaire - Driving (CFQ), Attention-Related Driving Errors Scale (ARDES), Mindfulness Attention Awareness Scale (MAAS), and Aggressive Driving Behavior Questionnaire (ADBQ). No measure was significantly related to participant demographics. Brief performance measures of cognition administered by telephone, including the Montreal Cognitive Assessment (MoCA) Blind and oral Trails A and B, were mostly uncorrelated with the self-reported driving and attention measures. One exception was the MoCA word recall and DRAS, r(106) = -.224, p=.02, in which poorer memory was associated with greater driver avoidance. These findings are noteworthy for 1) a lack of correlation between self-reported measures of driver attention and either age or cognition, 2) no gender differences found in measures of driver behavior, and 3) in a sample pre-screened for cognitive impairment, the word recall was significantly related to older adults’ self-reported driver avoidance behaviors.

